# CXCL10 could be a prognostic and immunological biomarker in bladder cancer

**DOI:** 10.1007/s12672-024-00982-6

**Published:** 2024-05-08

**Authors:** Tao Yin, Shuanzhu Mou, Haiyu Zhang, Ying Dong, Bing Yan, Weisheng Huang, Yuhan Liu, Hongbing Mei

**Affiliations:** 1 Department of Urology, The First Affiliated Hospital of Shenzhen University, Shenzhen Second People’s Hospital, Shenzhen University, Shenzhen, China; 2 Shenzhen University Medical College, Shenzhen, China; 3 Shenzhen Second People′s Hospital, Clinical Medicine College of Anhui Medical University, Shenzhen, China; 4 Guangdong Key Laboratory of Systems Biology and Synthetic Biology for Urogenital Tumors, The First Affiliated Hospital of Shenzhen University, Shenzhen Second People’s Hospital, Shenzhen, China

**Keywords:** CXCL10, Prognostic, Immunological, Biomarker, Bladder cancer

## Abstract

**Introduction:**

As proteins that promote immune cell differentiation, chemokines have attracted great interest regarding their role in anti-tumor immune responses within the cancer environment. However, the exact role of CXCL10, a chemokine, in bladder cancer (BLCA) is still not fully elucidated.

**Method:**

In the present study, we employed bioinformatics approaches to examine the expression pattern, prognostic value, and immune infiltration of CXCL10 in BLCA. Furthermore, we focused on examining the impact of CXCL10 on immune therapy in BLCA. Additionally, we validated the expression of CXCL10 in various BLCA cell lines using PCR techniques.

**Results:**

We observed an upregulation of CXCL10 in BLCA tissues as well as in different cell lines. Additionally, upregulation of CXCL10 indicates a better prognosis for BLCA patients. ESTIMATE and CIBERSORT algorithms suggest that CXCL10 is closely associated with the immune microenvironment of BLCA. Through multiple immune therapy cohorts, we also identified that CXCL10 has shown promising predictive value for assessing the efficacy of immune therapy in in BLCA.

**Conclusion:**

Our study indicates that CXCL10 has the potential to serve as a favorable prognostic factor and is strongly associated with immune infiltration in BLCA.

**Graphical Abstract:**

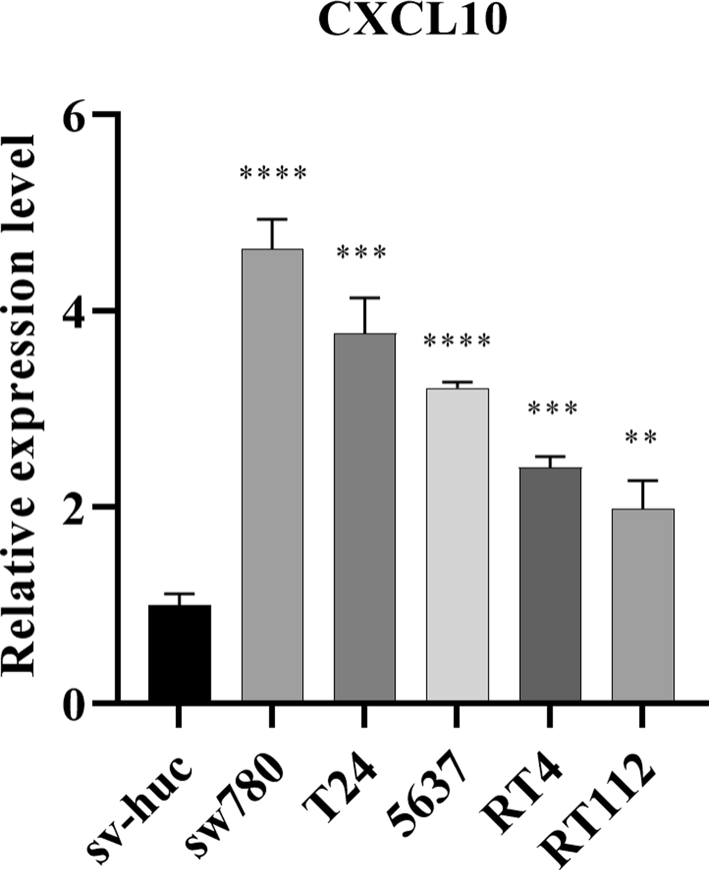

**Supplementary Information:**

The online version contains supplementary material available at 10.1007/s12672-024-00982-6.

## Introduction

Immune checkpoint inhibitors (ICIs) have become a crucial component in the treatment of bladder cancer (BLCA). ICIs enhance the ability of T cells to attack tumors by blocking immune inhibitory pathways such as PD-1, PD-L1, or CTLA-4. However, the response to ICIs varies significantly among BLCA patients, with only a small percentage of patients benefit from these agents. Screening for potential beneficiaries is an essential path for the development of precision medicine. Encouragingly, some molecular markers, such as chemokines, may serve as predictors of effective immune response in BLCA [1–5]. In addition, the development of interventions targeting chemokines may help enhance the effectiveness of immune therapy for BLCA.

As a secreted chemokine, C-X-C motif Chemokine Ligand 10 (CXCL10) is primarily produced by fibroblasts, cancer cells, endothelial cells, and monocytes in response to IFN-γ secretion [6]. Through binding to its unique receptor, CXC chemokine receptor 3 (CXCR3), CXCL10 exhibits pleiotropic functions in tumor biology [7]. Specifically, the CXCL10-CXCR3 axis can regulate immune cell activation, differentiation and migration to promote anti-tumor immunity through paracrine signaling. Conversely, tumor-derived CXCL10 can interact with CXCR3 to induce tumor cell proliferation, angiogenesis, and other pro-tumorigenic effects [6, 8–10]. CXCL10 has been demonstrated to coordinate anti-tumor immunity in BLCA. In the study by Tian et al., an immune signature containing CXCL10 demonstrated significant correlation with the immune microenvironment of BLCA, offering a new perspective for immunotherapy of BLCA [11]. In the healthy bladders of mice, Seow et al. found that the use of Bacillus Calmette-Guerin (BCG) alone significantly upregulated CXCL10 [12]. Conversely, in human bladder tissues, Muthuswamy et al. found that the use of BCG alone did not result in upregulation of CXCL10. However, the combination of BCG with IFNα and poly-I was found to be effective in enhancing CXCL10 in BLCA tissues and promoting cytotoxic T lymphocytes (CTLs) infiltration [13]. Ibrahim and colleagues conducted more in-depth research and made similar findings: the use of PGE2 antagonists alone, and the combination with BCG and indomethacin, can selectively enhance the chemoattractant factor CXCL10 for CTLs, achieving immune reprogramming in BLCA [14]. Additionally, research from Japanese scholars indicates that neutrophils expressing MHC class II and CXCL10 internally suggest a state of BCG-induced anti-tumor activity in BLCA [15]. Although the above studies suggest the potential of CXCL10 as a biomarker for immunotherapy in BLCA, overall, there is still a lack of research exploring the relationship between CXCL10 and recent immunotherapies, such as ICIs, in BLCA.

In the present research, we utilized multiple public databases to explore the expression patterns, prognostic value, and genomic instability of CXCL10 in BLCA. Moreover, we conducted an investigation into the role of CXCL10 in the BLCA immune microenvironment and its impact on immunotherapy. Our study provides new insights into the potential application of immunotherapy for BLCA.

## Methods

### Data acquisition and processing

The transcriptomic data and clinical information of CXCL10 were obtained from the Cancer Genome Atlas (TCGA) database, which can be accessed at https://tcga-data.nci.nih.gov/tcga/. The RNA sequencing data was normalized to transcripts per million (TPM). In addition, datasets including Imvigor210, GSE70691, GSE13507, and GSE39281 were also obtained for further analysis. The expression values were detected and performed by log2 transformation via R package “limma” (Version 3.56.2) [16].

### CXCL10 expression analysis

First, CXCL10 expression values were merged with clinical information from TCGA. Then, based on clinical information, the expression patterns of CXCL10 among different clinical subgroups were explored.

### Overall survival analysis

In the three datasets (TCGA-BLCA dataset, Imvigor 210, GSE70691), the expression levels of CXCL10 were merged with patient survival status, and survival time, respectively. Next, based on the median expression level of CXCL10 in the BLCA datasets, the samples were categorized into two groups, namely high- and low-expression groups of CXCL10. The “survival” (Version 3.5-7) and “survminer” (Version 0.4.9) packages available in the R software were performed to analyze the survival outcomes of CXCL10 between the two groups and generate survival curves.

### Genomic instability analysis

The genomic mutations of CXCL10 in different cancers were analyzed using cBioPortal (https://www.cbioportal.org/). The gene mutations, chromosome gain/loss between CXCL10 subgroups were explored in TCGA-BLCA cohort. The Microsatellite instability (MSI) data was assessed from the previous study [17]. Tumor mutation burden (TMB) scores were calculated using the mutation information of TCGA-BLCA samples by applying the R package “maftools” (Version 2.16.0) [18]. Differences of MSI and TMB in different CXCL10 expression subgroups were explored.

### TME analysis

First, differential expressed genes were screened based on the median CXCL10 expression by setting |log2Fold Change = 1| and p < 0.05. Then, the differentially expressed genes were analyzed by gene ontology (GO) and the kyoto encyclopedia of genes and genomes (KEGG) analyses. Moreover, the ESTIMATE algorithm was used to study the relationship between CXCL10 and immune infiltration score. Using R software, the CIBERSORT algorithm is run to evaluate the composition of immune cell types in each tumor sample in the form of proportions. The “limma” (Version 3.56.2) and “vioplot” (Version 0.4.0) packages in R software are used to analyze whether there are differences in the abundance of different immune cell types between the high CXCL10 expression group and the low CXCL10 expression group, and generate violin plots. The expression of immune checkpoints in the CXCL10 subgroup was also studied.

### Immunotherapy response analysis

The relationship between immune therapy response and CXCL10 expression was explored using four datasets (Imvigor210, Lauss, Kim, and Hwang cohort). Additionally, the Kaplan–Meier curve was employed to study the association between patient survival and CXCL10 expression in different immune therapy cohorts. The predictive performance of CXCL10 for immune therapy response was analyzed using ROC curves (package pROC, Version 1.18.4).

### qRT‑PCR

TRIzol was used to extract cellular RNA. The primers used were listed as follows: CXCL10: 5′-GTGGCATTCAAGGAGTACCTCTGATGGCCTTCGATTCTGGATT-3′; GAPDH: 5′-AGGGGAGATTCAGTGTGGTGGGCCTCCAAGGAGTAAGACC-3′. Analyses were conducted in triplicate.

### Statistical analysis

Differential expression analysis of CXCL10 in BLCA was performed by Kruskal–Wallis and Wilcoxon tests. Survival analysis of CXCL10 in BLCA was conducted by the log-rank test. The analysis of CXCL10's predictive ability for immune therapy response in BLCA was quantified using ROC curves. The correlation between CXCL10 and immune infiltration levels was investigated using the Spearman correlation coefficient. A p-value less than 0.05 was considered statistically significant.

## Results

### Expression analysis and survival analysis

**Fig. 1:**
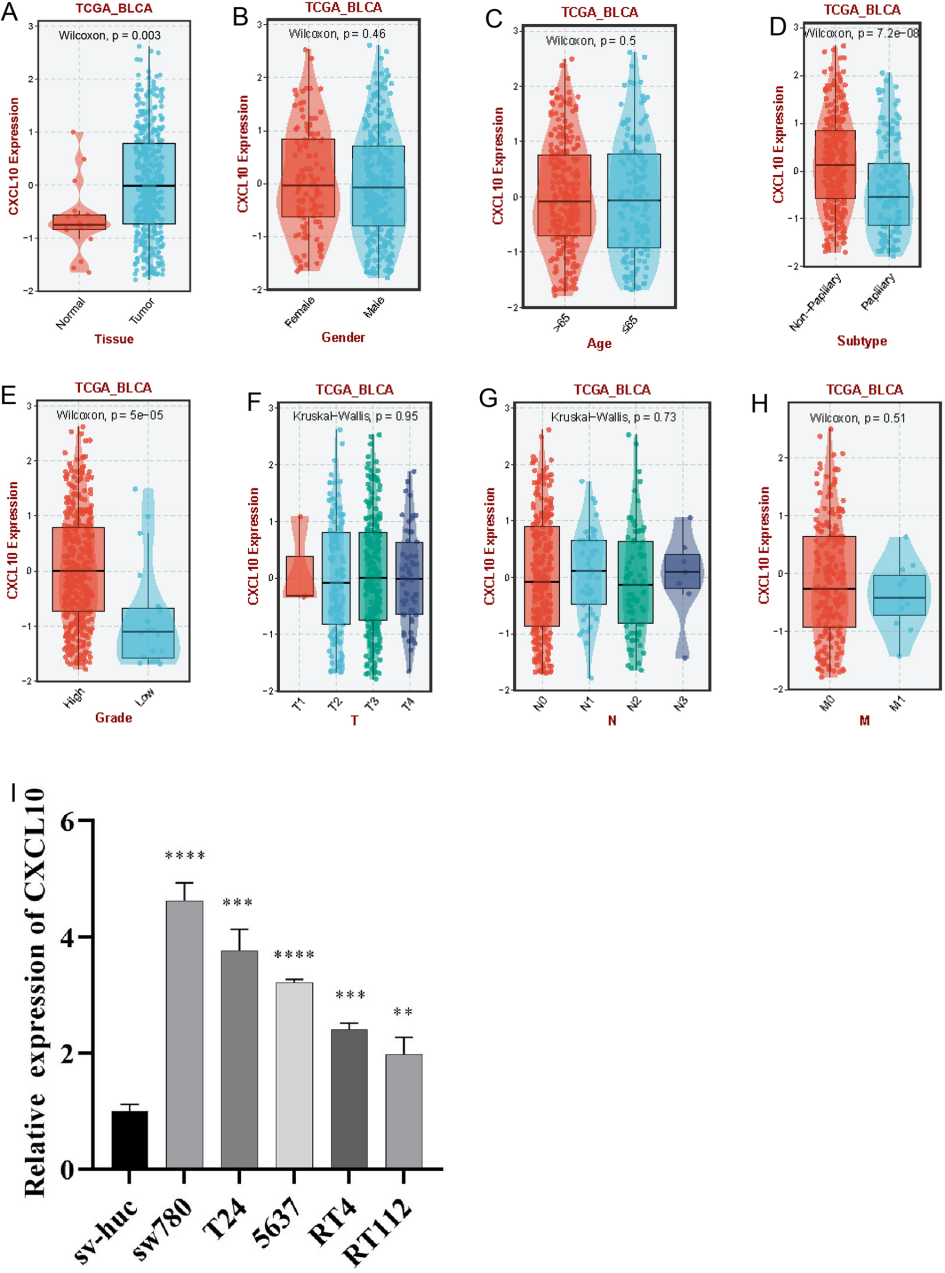
The expression of CXCL10 in BLCA. Expression of CXCL10 in different **A** tissues, **B** genders, **C** age, **D** subtypes, **E** grades, **F** T stage, **G** N stage, **H** M stage, and **I** BLCA cell lines

**Fig. 2:**
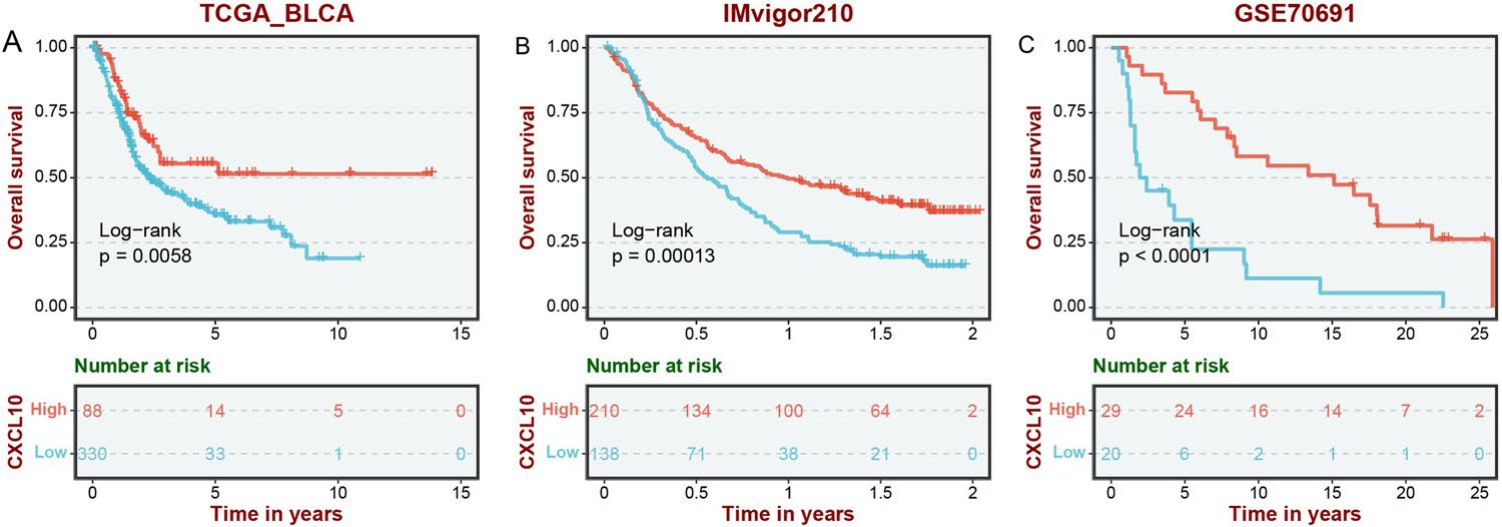
The overall survival rate of BLCA patients in CXCL10 subgroups. **A** TCGA-BLCA cohort. **B** IMvigor 210 cohort. **C** GSE70691 cohort

To obtain the expression pattern of CXCL10 in BLCA, we conducted a comprehensive expression analysis that incorporated different clinical variables from TCGA-BLCA cohort. It was observed that CXCL10 was significantly upregulated in tumor tissues (Fig. 1A; p = 0.003). Upregulation of CXCL10 expression was also observed in non-papillary tumors (Fig. 1D; p = 7.2e−08) and in higher grades (Fig. 1E; p = 5e−05). However, no statistically significant differences were observed with regards to gender (Fig. 1B, p = 0.46), age (Fig. 1C, p = 0.5), or T (Fig. 1F; p = 0.95), N (Fig. 1G; p = 0.73), and M staging (Fig. 1H, p = 0.51). Furthermore, we examined the impact of CXCL10 on the prognosis of BLCA patients. Analysis of TCGA-BLCA, Imvigor210, and GSE70691 datasets revealed that the survival analysis of CXCL10 in BLCA showed statistically significant differences (Figs. 2A–C) (log-rank test, p < 0.05), suggesting that patients with high CXCL10 expression may have a better prognosis.

In addition, we verified the expression of CXCL10 in different human bladder cancer cells by qRT‑PCR. The findings revealed a significant upregulation of CXCL10 in a variety of bladder cancer cells, including sw780, T24, 5637, RT4, and RT112, compared with normal bladder cell(Sv-huc) (Fig. 1I, all p < 0.05 Table 1).

### CXCL10 is involved in the genomic instability of BLCA

**Fig. 3:**
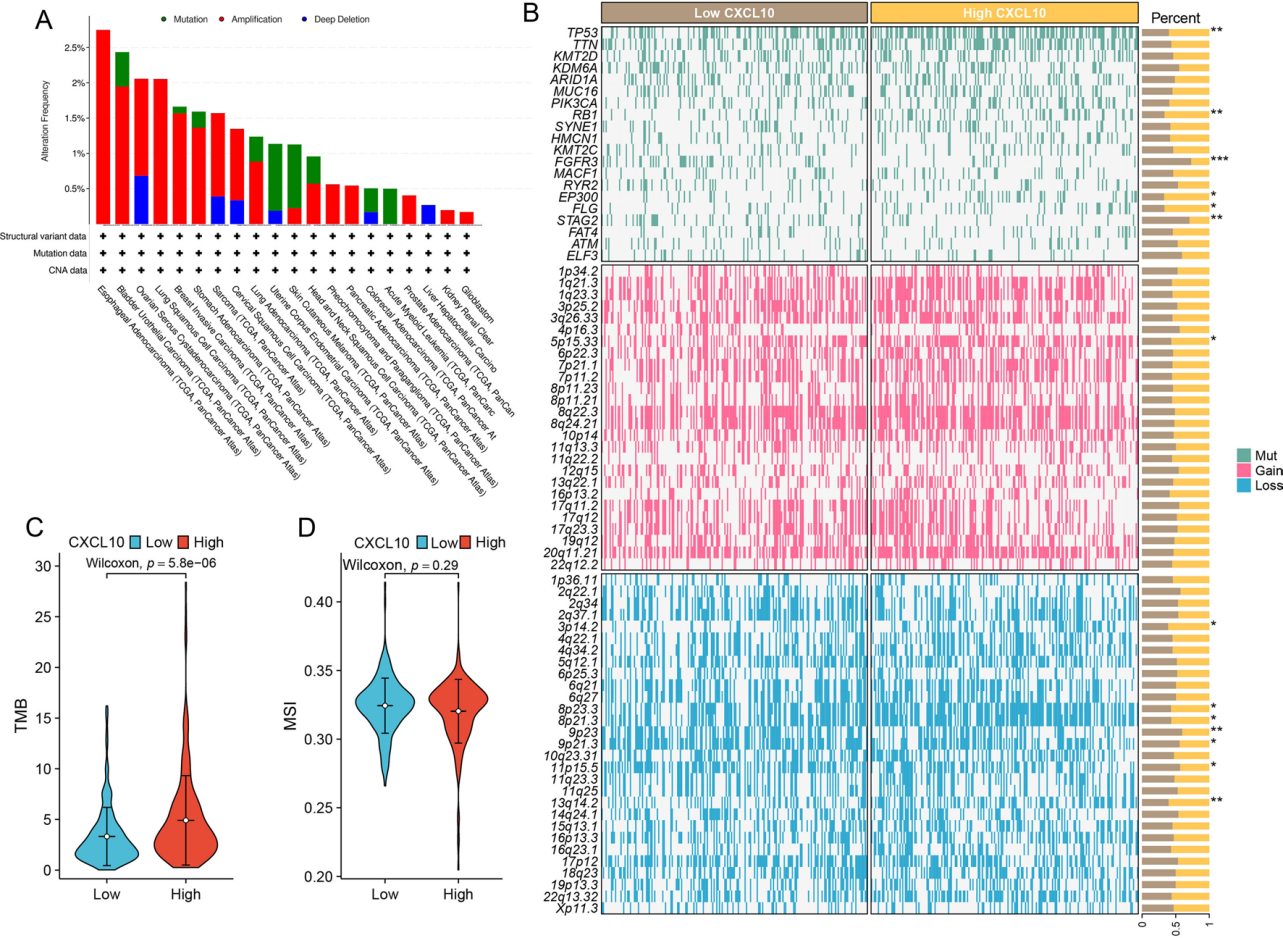
CXCL10 is involved in the genomic instability of BLCA. **A** Gene alterations of CXCL10 in BLCA mainly included mutation, amplification, and deep deletion. **B** Gene mutation and chromosome gain/loss between CXCL10 subgroups. The **C** TMB and **D** MSI between CXCL10 subgroups

Genomic instability is known to play a critical role in the development of cancer [19]. To understand whether CXCL10 is associated with genomic instability in BLCA, we first conducted an analysis using cBioPortal. The results showed that the types of gene alterations of CXCL10 in BLCA mainly included mutation, amplification, and deep deletion (Fig. 3A). Furthermore, we found that patients with high CXCL10 expression in TCGA-BLCA have a higher tumor mutation burden (TMB) (Fig. 3C, p < 0.001), however, our analysis indicated no significant difference in microsatellite instability (MSI) within the CXCL10 subgroups (Fig. 3D, p = 0.29). In addition, between the high and low CXCL10 expression groups, several genes (TP53, RB1, FGFR3, EP300, RYR2, FLGz) exhibited significantly different mutation frequencies. In the high CXCL10 group, there was a significant gain in chromosome 5p15.33, while 3p14.2, 8p23.3, 8p21.3, 9p23, 9p21.3, 11p15.5, and 13q14.2 showed significant loss (Fig. 3B, p < 0.05).

### Functional enrichment analysis

**Fig. 4:**
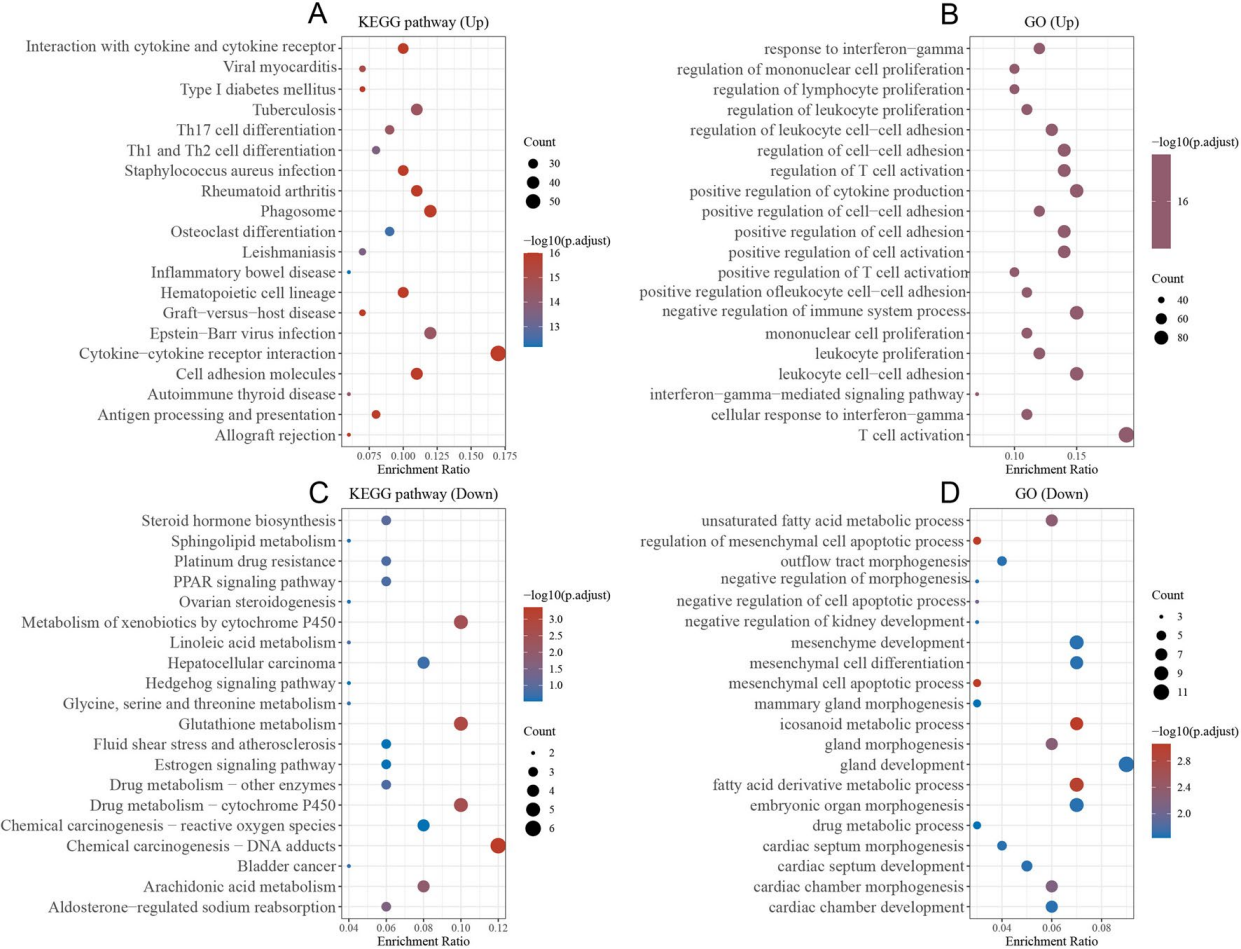
Functional analysis of CXCL10 in BLCA. **A**, **C** KEGG. **B**,** D** GO

To further explore the role of CXCL10 in BLCA, we grouped genes based on CXCL10 expression values and explored differentially expressed genes. We then subjected these genes to gene ontology (GO) and Kyoto Encyclopedia of Genes and Genomes (KEGG) analyses to determine their biological functions. The results revealed that in the group with high expression of CXCL10, several immune pathways were enriched, including those related to immune response and the generation of immune cells (Fig. 4A, B). These pathways are essential for immune responses. On the other hand, the group with low expression of CXCL10 was primarily enriched in processes such as substance metabolism and energy conversion (Fig. 4C, D). It is thus inferred that CXCL10 plays an important role in the immune processes of BLCA and may be involved in the progression of BLCA through multiple immune pathways.

### TME analysis

**Fig. 5:**
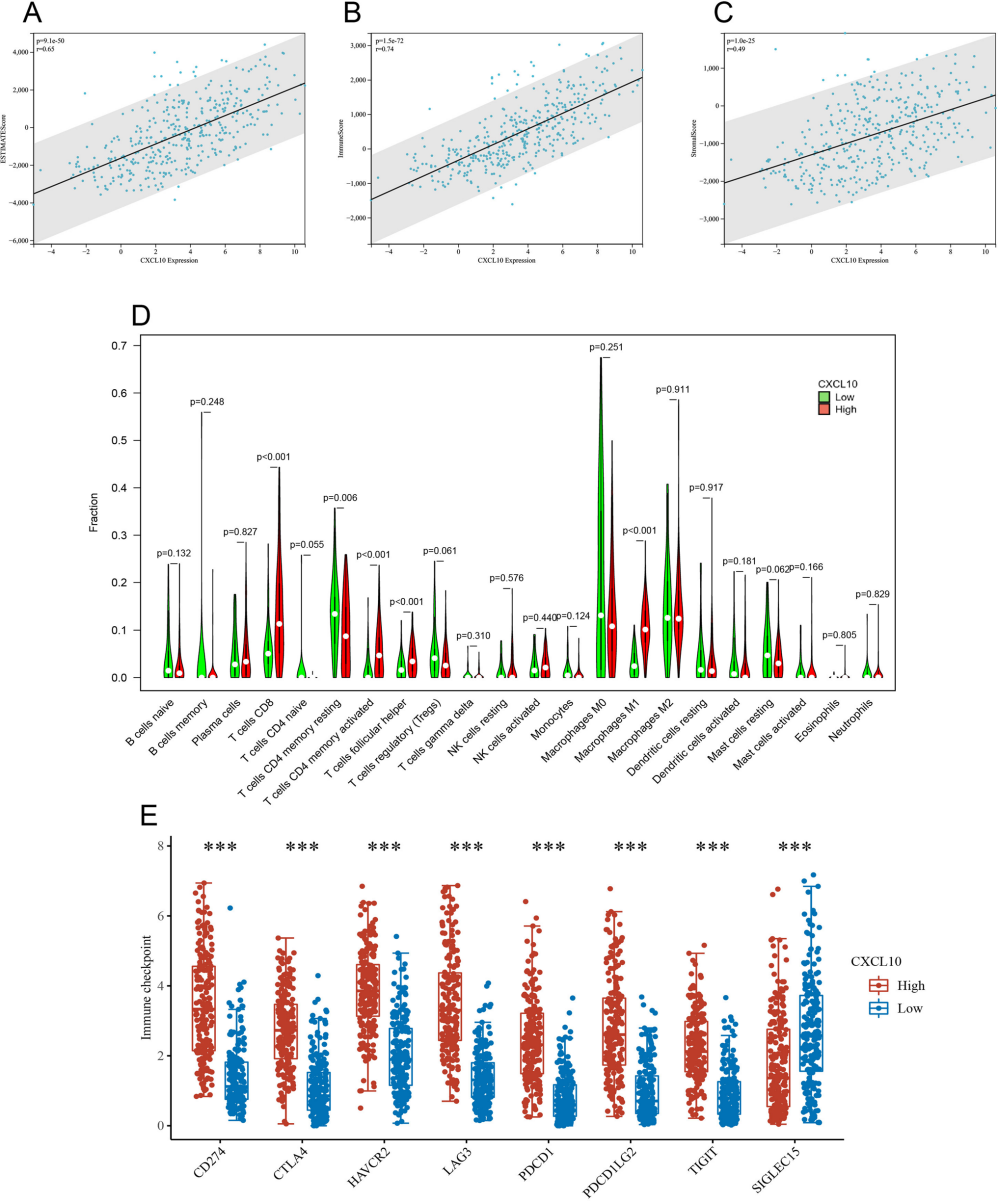
CXCL10 mediates the TME of BLCA patients. **A**–**C** ESTIMATE analysis of CXCL10 in BLCA. **D** CIBERSORT. **E** Immune checkpoints in CXCL10 subgroups

TME comprises various components, including tumor cells, immune cells, inflammatory cells, stem cells, vascular system, fibrous tissue, and extracellular matrix (ECM), and TME has been demonstrated to play a crucial role in the occurrence and progression of BLCA [20]. Given that CXCL10 may influence BLCA through multiple immune processes, we conducted an analysis of the role of CXCL10 in the TME. Firstly, employing the ESTIMATE algorithm, we found a significant correlation between CXCL10 and both stromal score and immune score (Figs. 5A–C, all p < 0.05). Next, we explored the relationship between CXCL10 and different immune cells. The results showed that in the group with high CXCL10 expression, there were higher abundances of CD8 T cells, CD4 T cells, and M1 macrophages (Fig. 5D, p < 0.05), all of which play important roles in anti-tumor immune processes. In recent years, the effectiveness of immune checkpoint inhibitors in the field of immunotherapy has become increasingly evident. Therefore, we investigated the relationship between CXCL10 and different immune checkpoints. Surprisingly, the group with high CXCL10 expression exhibited significantly elevated levels of immune checkpoint expression, including CTLA4 and PD1 (Fig. 5E, all p < 0.001).

### Immunotherapy response analysis

**Fig. 6:**
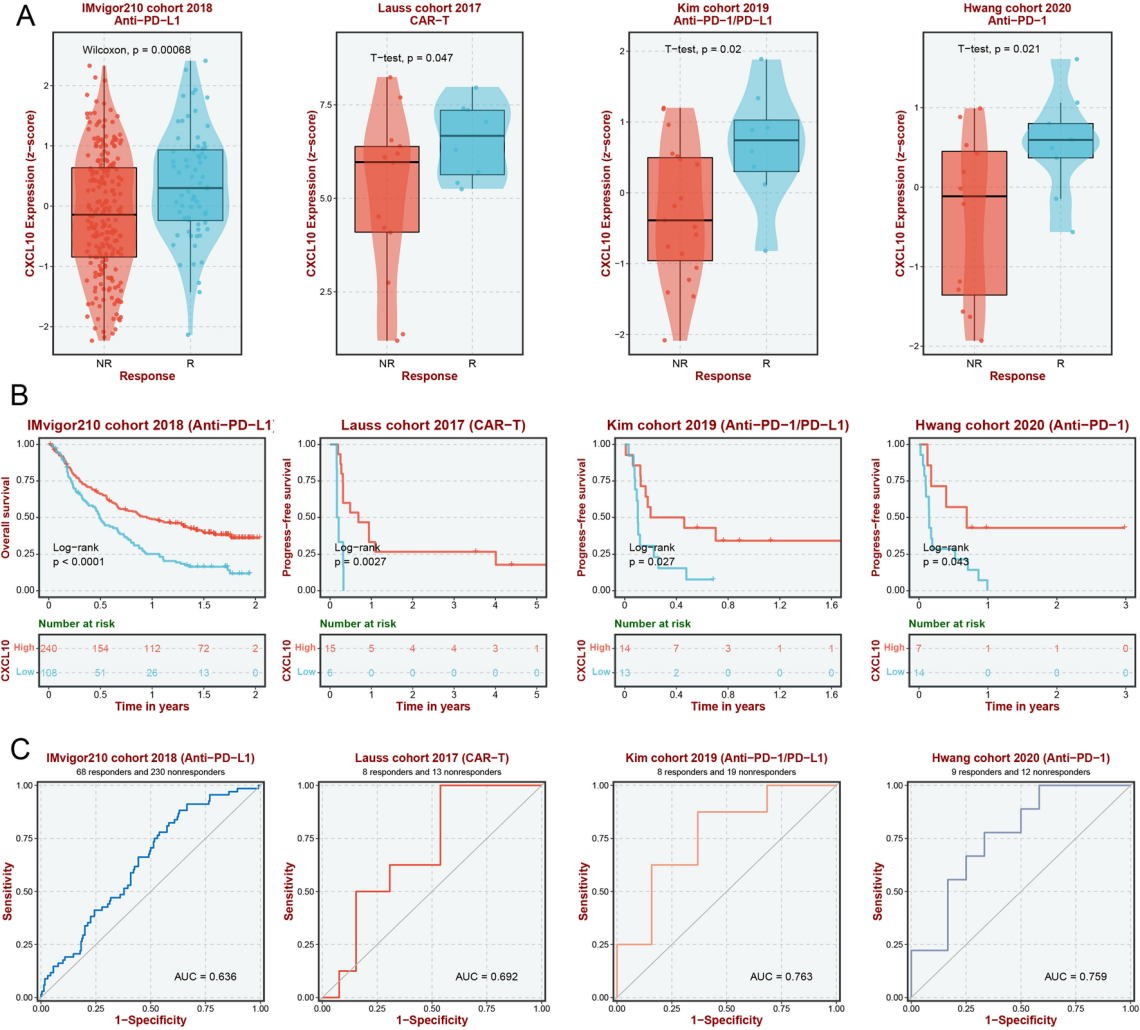
CXCL10 could predict the immune response of BLCA patients. **A** Immune response between CXCL10 subgroups. **B** Survival analysis of BLCA patients after receiving immune therapies between CXCL10 subgroups. **C** ROC curves of CXCL10 predicting the immune responses

The response to immunotherapy varies among different BLCA patients. Predicting the response to immunotherapy based on potential biomarkers can help achieve personalized treatment and optimize resources utilization [21]. Combining multiple BLCA immunotherapy cohorts, we focused on exploring the impact of CXCL10 on immune therapy response. Firstly, in multiple immunotherapy cohorts (IMvigor210, Lauss, Kim, and Hwang cohort), the immune therapy-responsive group exhibited higher expression of CXCL10 compared to the non-responsive group (Fig. 6A, all p < 0.05). Moreover, in patients who received immune therapy, including anti-PD-1/PD-L1 and CAR-T therapies, the high expression group of CXCL10 showed better overall survival and progression-free survival (Fig. 6B, all p < 0.05). CXCL10 also demonstrated a certain level of accuracy in predicting the response of BLCA patients to immune therapy (IMvigor210 cohort: Lauss, Kim, and Hwang cohort) (Fig. 6C).

## Discussion

Due to advances in treatment methods, the prognosis of BLCA patients is gradually improving [22]. However, existing treatments are still ineffective for some refractory cases, and tumor metastasis and invasion remain huge challenges for treatment. Encouragingly, biomarkers play an important role in assessing the prognosis of BLCA patients and selecting treatment methods. Thus, more biomarkers for BLCA need to be explored. In addition, recently, novel treatment options such as PD-1/PD-L1 inhibitors for bladder cancer have continued to emerge, but immunotherapy is still in its infancy and more efforts are needed [23].

In this study, we provided evidence of high expression of CXCL10 in BLCA. Furthermore, our survival analysis indicated that high CXCL10 expression was associated with a favorable prognosis. These findings reveal the clinical value of CXCL10 as a prognostic factor in BLCA. Additionally, we found that CXCL10 was involved in genomic instability in BLCA. Genomic instability refers to the tendency of genomic changes and abnormalities within cells, such as chromosomal instability, accumulation of mutations, gene defects, and functional abnormalities. Genomic instability frequently occurs in tumors and is closely associated with tumor development and progression [24]. Studying genomic instability is of great significance for a deeper understanding of tumor occurrence and treatment. Particularly, high expression CXCL10 group exhibited higher TMB levels, which is also a biomarker for predicting immune therapy response [25].

Further functional enrichment analysis revealed that genes in the CXCL10 high-expression group were enriched in pathways related to immune response and immune cell generation. We found that CXCL10 is closely associated with upregulated cytokine-cytokine receptor interaction pathway. As a secreted protein or peptide, cytokines transmit signals between cells and regulate biological processes such as immune and inflammatory responses [26]. Furthermore, in the GO analysis, CXCL10 is closely related to T cell activation [27]. This also indicates the close relationship between CXCL10 and the immune microenvironment of bladder cancer (BLCA). Therefore, we used ESTIMATE to calculate the relationship between CXCL10 and the immune microenvironment of BLCA. Not surprisingly, the expression of CXCL10 showed a strong correlation with immune scores and stromal scores. Based on this, we further investigated the correlation between CXCL10 and various immune cells using CIBERSORT. We found that the levels of CD8 + T cells, CD4 + T cells, and M1 macrophages were significantly higher in the CXCL10 high-expression group compared to the low-expression group. T cells are a crucial cell type within the immune system, playing a key role in regulating and mediating immune responses. T cells, particularly CD8 + T cells, can recognize and attack abnormal tumor cells through the interaction of their specific T cell receptors (TCRs) with antigens present on the surface of these cells [28]. In addition to CD8 + T cells, CD4 + T cells also involve in the tumor immune microenvironment by activating and regulating the functions of other immune cells such as CD8 + T cells and macrophages. They enhance the anti-tumor immune response by releasing cytokines, such as tumor necrosis factor-alpha (TNF-α) and interferon-gamma (IFN-γ) [29]. In the group with high expression of CXCL10, we observed a significant upregulation of M1 macrophage abundance. In the TME, M1 macrophages are a subtype of macrophages that possess anti-tumor activity. M1 macrophages can participate in anti-tumor immune responses through multiple mechanisms, such as recruiting other immune cells and directly attacking tumor cells. Interestingly, M1 macrophages can also suppress immune evasion of tumor cells by regulating the expression of immune checkpoint molecules such as PD-L1. In addition to its strong correlation with immune cells, we also discovered that in the high expression group of CXCL10, there was a significant upregulation in the expression of various immune checkpoints. This suggests that the use of immune checkpoint inhibitors may be more effective in BLCA patients with high expression of CXCL10. Taken together, these results suggest that CXCL10 is involved in the regulation of cellular and humoral immunity in BLCA, and it may affect the tumor microenvironment of BLCA through multiple immune pathways.

A improved understanding of the TME in BLCA can contribute to achieving better immunotherapy outcomes. Given that our results indicate the involvement of CXCL10 in the regulation of the BLCA immune microenvironment, we further investigated the impact of CXCL10 on immunotherapy for BLCA. We found that in multiple immune therapy cohorts, such as Imvigor 210, an immune therapy cohort for BLCA, high expression of CXCL10 indicates better immune therapy outcomes, including the popular immune checkpoint inhibitor therapy and adoptive cell therapy. We speculate that this may be due to higher immune cell infiltration in the TME of patients with high CXCL10 expression, such as CD8 + T cells, M1 macrophages and CD4 + T cells. When immune checkpoint inhibitors are used in patients with high CXCL10 expression, they block inhibitory receptors on the surface of T cells, which triggers higher levels of immune cells to recognize and kill tumor cells, resulting in higher response rates and improved efficacy of cancer treatment. In response, higher CXCL10 expression is associated with better overall survival and PFS in patients receiving immune therapy. This is attributed to the activation or enhancement of the body's immune system by immune therapy to suppress tumor growth and metastasis, leading to improved prognosis for patients. In addition, we also found that CXCL10 has certain accuracy in predicting the effectiveness of immune therapy. Due to the heterogeneity of malignant tumors, not all BLCA patients can benefit from immune therapy to the same extent. Some patients may have a strong response to immune therapy, while others may have no obvious effect. Given that CXCL10 predicts the effectiveness of immune therapy, it can help doctors choose the most suitable treatment plan for patients and achieve personalized treatment. Taken together, we believe that CXCL10 is a potential biomarker for predicting the immune therapeutic response of BLCA.

## Conclusion

In this study, we confirmed the significant upregulation of CXCL10 in BLCA through the use of multiple public databases and qPCR. We also found that high expression of CXCL10 is associated with a favorable prognosis in BLCA and that CXCL10 may regulate the immune microenvironment of BLCA. In conclusion, CXCL10 is a promising biomarker for predicting a favorable prognosis and associated with immune infiltration in BLCA, which can effectively predict the response to immune therapy in BLCA.

### Supplementary Information

Supplementary file 1 (XLSX 14 KB)

Supplementary file 2 (XLS 57 KB)

## Data Availability

The original contributions presented in the study are included in the article/Supplementary Material. Further inquiries can be directed to the corresponding author.

## Abbreviations

BLCA: Bladder urothelial carcinoma
TCGA-BLCA: The Cancer Genome Atlas-Bladder Cancer
GEO: Gene-Expression Omnibus
RT-qPCR: Real-time quantitative polymerase chain reaction
GO: Gene ontology
KEGG: Kyoto Encyclopedia of Genes and Genomes
GAPDH: Glyceraldehyde-3-phosphate dehydrogenase
MSI: Microsatellite instability
TMB: Tumor mutation burden
TME: Tumor microenvironment
